# COL4A2 enhances thyroid cancer cell proliferation through the AKT pathway

**DOI:** 10.32604/or.2024.047382

**Published:** 2024-08-23

**Authors:** LIANG HE, WEI HAN, KAI YUE, XUDONG WANG

**Affiliations:** 1 Department of Maxillofacial and Otorhinolaryngological Oncology, Tianjin Medical University Cancer Institute and Hospital, Key Laboratory of Basic and Translational Medicine on Head & Neck Cancer (Tianjin), Key Laboratory of Cancer Prevention and Therapy, Tianjin Cancer Institute, National Clinical Research Center for Cancer, Tianjin, 300060, China; 2 Department of Thyroid & Breast Surgery, Luoyang Central Hospital, Luoyang, 471000, China; 3 Department of Otorhinolaryngological Surgery, Zhengzhou Central Hospital, Zhengzhou, 450000, China

**Keywords:** Thyroid cancer (THCA), Proliferation, COL4A2, AKT pathway, Biomarker cancer progression

## Abstract

**Objectives:**

Thyroid cancer (THCA) is the most common malignant tumor in endocrine system and the incidence has been increasing worldwide. And the number of patients dying from THCA has also gradually risen because the incidence continues to increase, so the mechanisms related to effective targets is necessary to improve the survival. This study was to preliminarily investigate the effects of the COL4A2 gene on the regulation of thyroid cancer (THCA) cell proliferation and the associated pathways.

**Methods:**

Bioinformatics analysis revealed that COL4A2 was closely associated with cancer development. COL4A2 expression in THCA tissues was analyzed using immunohistochemistry, and survival information was determined via Kaplan‒Meier curves. The expression of COL4A2 and AKT pathway-related genes were analyzed using qPCR and western blot analyses. Colony formation as well as CCK-8 assays exhibited the cell proliferation level and cell activity, respectively. Downstream of COL4A2 was identified by Gene set enrichment analysis (GSEA). The effects of the COL4A2 and AKT pathways on THCA tumor growth *in vivo* were determined using a mouse model.

**Results:**

Bioinformatics analysis exhibited that COL4A2 plays a significant role in cancer and that the AKT pathway is downstream of COL4A2. THCA patients with high COL4A2 expression had shorter recurrence-free survival. Upregulation of COL4A2 gene expression in 2 THCA cell lines promoted tumor cell growth and activity. The use of AKT pathway blockers also restrained the growth and activity of the 2 THCA cell lines. The use of AKT pathway blockers reduced tumor volume and mass and prolonged mouse survival.

**Conclusions:**

COL4A2 can promote the growth as well as development of THCA through the AKT pathway and COL4A2 could be used as a target for THCA.

## Introduction

Thyroid cancer (THCA) is the most common malignant tumor and accounts for approximately 95% of tumors in endocrine system [1]. Recently, the incidence of THCA has been increasing worldwide. Globally, thyroid cancer has jumped to 4th place in cancer incidence [1]. In Korea, thyroid cancer is one of the most prevalent malignant tumors in women and a serious threat to human health [2]. In China, the incidence of THCA has also been increasing annually, with the standardized incidence rate increasing from 1.4 per 100,000 person-years in 1990 to 14.65 in 2016; now, THCA has become the 7th most prevalent malignant tumor in China, with an increasing incidence rate, especially in the female population [3]. According to statistics from 2021, THCA incidence in females has increased from 7th to 3rd place. As the THCA incidence continues to increase, the number of patients dying from thyroid cancer has also gradually risen. Although the survival rate of Chinese thyroid cancer patients has risen to 84.3% through precise preoperative diagnosis, standardized surgical treatment, the application of new technologies, and standardized and individualized postoperative follow-up, there is still a significant gap between the survival rate in China (84.3%) and the survival rate in major developed countries (98%) [1].

Collagen type IV (Collagen IV) is known to be essential for the physical integrity and multiple signaling functions of muscle tissue [4]. In contrast, COL4A2 is a key member of the COL4 family and has a variety of critical functions [5]. Previous studies have reported cases of COL4A2 gene mutations in patients with severe brain injury [6]. Moreover, the role of COL4A2 in a variety of cancers has also been revealed [7]. COL4A2 is widely expressed in different tumor tissues, and abnormally high COL4A2 expression has been found in cancers such as hepatocellular carcinoma, breast cancer and glioma. A previous microarray analysis showed that COL4A2 is a potential biomarker of poor prognosis in Asian cancer patients, but this needs to be further investigated [8]. In addition, its possible pathways of action in cancer tissues are not fully understood.

The PI3K/AKT axis regulates the proliferation and cell cycle of various tumor cells, promotes tumor angiogenesis, contributes to tumor invasion and metastasis, regulates apoptosis and autophagy, and participates in tumor drug resistance, which in turn affects tumor development [9]. Moreover, some studies have shown that miRNAs can regulate tumor angiogenesis by affecting COL4A2 through the PI3K/AKT pathway [10]. Therefore, elucidating the mechanisms related to the COL4A2 and AKT pathways in THCA patients is necessary to improve the survival of these patients.

Here, we first screened the THCA-related gene COL4A2 by bioinformatics analysis and explored the effect of the COL4A2 gene on the regulation of THCA cell proliferation. Interestingly, COL4A2 was high expression in THCA cells, and its expression was significantly negatively correlated with overall survival. The results indicated that COL4A2 could enhance the growth and activity of THCA cells. Subsequently, we identified the AKT pathway as a potential downstream pathway by GSEA and demonstrated that COL4A2 could promote the growth of THCA through the AKT pathway by suppressing the AKT pathway. This study further supports the role of COL4A2 in THCA development.

## Materials and Methods

### Patients and samples from public databases

The gene expression profiles of GSE5364, GSE33630, and GSE58545 were obtained from the GEO database (http://www.ncbi.nlm.nih.gov/geo/). The GSE5364, GSE33630, and GSE58545 datasets were divided into gene sets using unsupervised cluster analysis based on the three dimensions of tumor size, Ki67 index, and survival time, and the gene sets were subsequently subdivided into modules on this basis. The modules were subsequently clustered into clusters that were found to be correlated with the MAPK signaling pathway and those with the most significant positive correlation with the MAPK pathway. Afterwards, the expression levels of the COL4A2 gene in each cluster were represented using a chordal plot, and the relative importance of the COL4A2 gene was explored via a contour plot of the clusters with the most significant positive correlation with the MAPK pathway. Biological processes associated with COL4A2 were explored by conducting a functional analysis of COL4A2 (FunctionalAnalysis_ORA_KEGG_COL4A2). Twenty-two pairs of samples were obtained from 22 patients diagnosed with THCA, and each pair of samples consisted of paired tumor tissue and normal tissue samples from the same patient. All applicable international, national, and/or institutional guidelines for the care and use of human specimens and animals were followed. We have used the samples from our hospital for the results, and the ethical approval number is bc2022100. The patient informed consent are not required because patients and samples from public databases.

### Mice, cell culture and reagents

Four-week-old female BALB/c nude mice were obtained from Beijing Vital River Co., China. The CAL-62 and BCPAP cell lines were obtained from ATCC. The cell culture media used were DMEM or RPMI 1640 (Gibco, California, USA) supplemented with 10% FBS at 37°C with 5% CO_2_. The *in vivo* study ethical approval number is IRM-DWLL-2023183. All animal assays were approved by the Institute of Radiation Medicine Ethics Committee, Chinese Academy of Medical Sciences.

The COL4A2 rabbit anti-human monoclonal antibody (ab251864, Thermo Fisher Scientific, USA; immunohistochemistry, 1:200 dilution; immunoblotting, 1:1000 dilution); goat anti-rabbit COL4A2 secondary antibody (ab256721, Wuhan Three Eagles, China; immunohistochemistry, 1:200 dilution; WB, 1:1000 dilution); GAPDH (Cell Signaling, USA); diaminobenzidine (Cell Signaling, USA); TRIzol Reagent (15596026, Invitrogen, USA); first strand cDNA synthesis kit (K1621, Thermo, USA); membrane-associated protein V-APC; and propidium iodide (PI) staining reagents (BioLegend, California, San Diego, USA). A RM2235 paraffin sectioning machine (Leica, Germany) and a BX51 microscope (Olympus, Japan) were used.

### Immunohistochemical staining

Formalin-fixed tumor tissues were routinely paraffin-embedded, after which the paraffin-embedded tissue sections were incubated at 75°C for approximately 30 min, deparaffinized with xylene solution, dehydrated through a gradient alcohol series, etc., and subsequently incubated with 2% BSA and incubated with an anti-MELK antibody overnight at 4°C. The sections were left for 1 h and washed twice with PBS for 2 min each time. The sections were incubated with secondary antibody for 1 h, washed and stained with diaminobenzidine for 5 min. Finally, the sections were sealed with neutral dendrimer.

### Stabilized cell lines

ShRNAs against COL4A2 in the PLKO-Puro lentiviral vector were purchased from Sangon. The main plasmid and helper plasmid were packaged into the virus using 293T cells as the host.

### Real-time quantitative PCR

Total RNA was extracted from the cells using RNAiso Plus (TaKaRa, Japan). The obtained total RNA was then reverse transcribed to obtain cDNA. The cDNA templates were subsequently analyzed using qPCR Master Mix (Biomake, USA).

**FIGURE A1:**
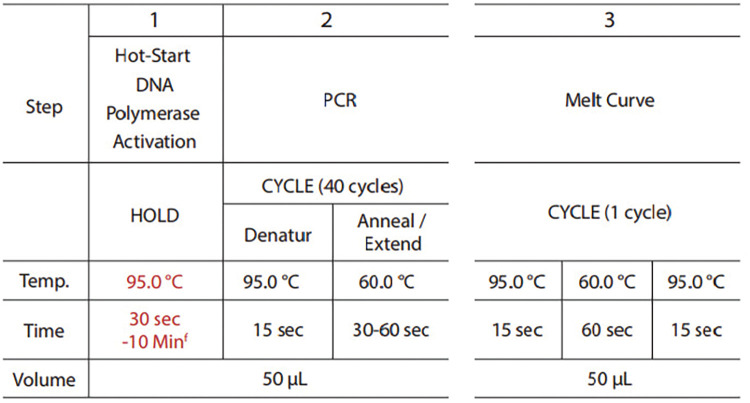
The specific qPCR procedure: The annealing temperature used for the primers, the number of PCR cycles and the volume of the reagent system in the specific qPCR procedure.

The primer sequences for GAPDH and COL4A2 are shown in Table 1. More information about the specific qPCR procedure, such as the annealing temperature used for the primers and the number of PCR cycles, is provided in Fig. A1.

**Table 1:** The primers sequence used in this study

Primer	GAPDH	COL4A2
Forward primer	5′-TGCACCACCAACTGCTTAGC-3′	5′-CGGAGTTTGTGGATCGGATA-3′
Reverse primer	5′-GGCATGGACTGTGGTCATGAG-3′	5′-CCCCCGTTACACTCGATAAA-3′

### Western blot

Proteins were separated by SDS‒PAGE on a 10% gel and then transferred. The PVDF membranes were incubated with 1:1000 dilutions of anti-COL4A2 and anti-GAPDH primary antibodies at 4°C overnight.

The PVDF membrane was then washed and incubated with a 1:1000 dilution of goat anti-rabbit COL4A2 secondary antibody, and the results were visualized using 1 mL of enhanced chemiluminescence (ECL) (A38555, Thermo Fisher Scientific, USA) Western blotting detection reagent after the PVDF membrane was incubated in the dark for 10 min. The grayscale values of the protein isolation bands were analyzed using Image-Pro Plus 5.0 software.

### Colony formation assay

The cells were diluted in 10% FBS, plated into 6-well plates (1000 cells/well) and incubated at 37°C in humid air with 5% CO_2_ for 14 days, fixed, and stained with 1 ml of 0.2% crystal violet for 20 min, and counted using a BX51 microscope.

### CCK-8 experiment

The cells were then diluted to a concentration of 50,000 cells/mL using serum-containing medium. 100 µL of fresh medium containing 10% CCK-8 was added to each well for 4 h. The OD450 value was measured.

### Gene set enrichment analysis (GSEA)

THCA samples were downloaded from the TCGA database. Gene set enrichment analysis (GSEA) was performed using the KEGG gene set collection to identify pathways that were differentially enriched between the high-and low-COL4A2 expression groups in THCA tissues. Pathways were considered significantly differentially enriched if they had an adjusted *p* value (FDR) < 0.05 and a positive enrichment score (ES).

### Mouse tumor growth assay

The Animal Ethics Committee of this study approved the use of BALB/c female nude mice (4 weeks old, weighing 18–22 g) for the relevant mouse experimental manipulations. All animal assays were approved by the Institute of Radiation Medicine Ethics Committee, Chinese Academy of Medical Sciences (Approval no. IRM-DWLL-2023183). The mice were housed at 20°C and 60% humidity, and provided a 12-h light/dark cycle. Twenty-four nude mice (n = 6, X4 groups) were randomly divided, and the remaining 40 nude mice (n = 10, X4 groups) were used for a parallel survival experiment. To detect tumor growth, 5 × 10^6^ primary CAL-62 cells or COL4A2-overexpressing cells were mixed with 100 µL of saline and injected subcutaneously into nude mice. All the mice received a single injection. Tumor volume was measured on day 6 after successful inoculation (day 0) and every 3 days thereafter. On the 27th day, the mice were sacrificed, and the tumors were isolated, photographed, weighed and counted. Moreover, in a parallel survival trial, the mice were kept until death, and Kaplan‒Meier curves were generated.

### Statistical analysis

The statistical data are presented as the mean ± standard deviation (SD) and were analyzed using GraphPad Prism 8.0.1 software. A two-tailed, unpaired *t-*test was used to compare the means of two samples. A χ^2^ test was used to compare the count data of the two groups of patients. *p* < 0.05 was considered to indicate statistical significance.

## Results

### COL4A2 was identified as a key gene associated with cancer development

**Figure 1:**
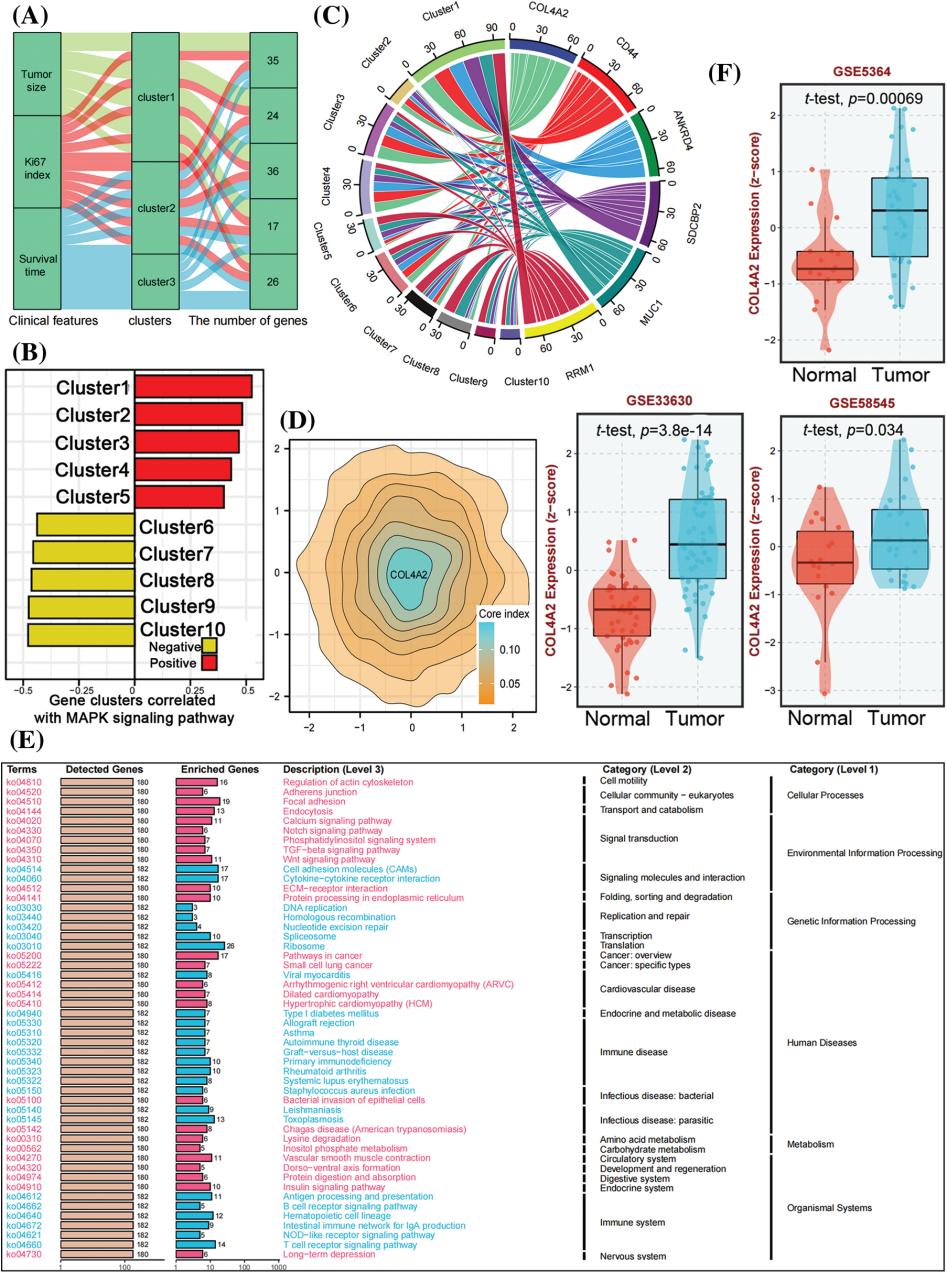
Identification of COL4A2 through bioinformatics analysis. (A) Sankey diagram showing that, through unsupervised clustering analysis of the TCGA (THCA), GSE5364, GSE33630, and GSE58545 datasets based on tumor size, Ki67 index, and survival time, the genes were divided into three gene sets, which were further subdivided into five modules. (B) A bidirectional bar chart was used to cluster the five modules into 10 clusters, and Pearson correlation analysis was performed with the MAPK signaling pathway (MAPK is a prominent pathway contributing to tumor proliferation promotion). Cluster 1 had the most significant positive correlation with the MAPK pathway. (C) Chord diagram visualizing the intensity of gene interactions in each cluster. COL4A2 had the most significant effect in cluster 1. (D) In the contour map, COL4A2 has the highest weight in cluster 1. (E) Functional analysis of ORA-KEGG_COL4A2 revealed that COL4A2 is involved in various biological processes and is closely related to the cancer progression. (F) The expression of COL4A2 in cancer and adjacent tissues in the GSE dataset. The data are expressed as the mean ± SD from 3 repeated experiments.

First, the GSE5364, GSE33630 and GSE58545 datasets were classified into three clusters of gene sets by unsupervised cluster analysis based on the three dimensions of tumor size, Ki67 index, and survival time and then subdivided into five modules, which were represented by Sankey diagrams (Fig. 1A). A total of 138 genes were identified. The five modules in Fig. 1A were then clustered into 10 clusters for correlation analysis (Pearson correlation) with the MAPK pathway (MAPK is a pathway that contributes very prominently to tumor proliferation); it was found that cluster 1 had the most significant positive correlation with the MAPK pathway and was represented as a bidirectional bar graph (Fig. 1B). The expression levels of the 10 clusters were specifically analyzed and represented as a chord plot (Fig. 1C), and COL4A2 was found to have the most significant intensity of action in cluster 1. Further exploration of COL4A2 expression revealed that COL4A2 expression was greatest in cluster 1 (Fig. 1D). COL4A2 is involved in the development of multiple cancers (Fig. 1E). Subsequently, after determining the expression of COL4A2 in tumor tissues and paracancerous tissues in the GSE5364, GSE33630, and GSE58545 cohorts, it was found that the expression of COL4A2 in cancer tissues was significantly greater than normal (Fig. 1F, *p* < 0.05).

### COL4A2 is highly expressed in THCA and negatively correlated with the prognosis of patients

**Figure 2:**
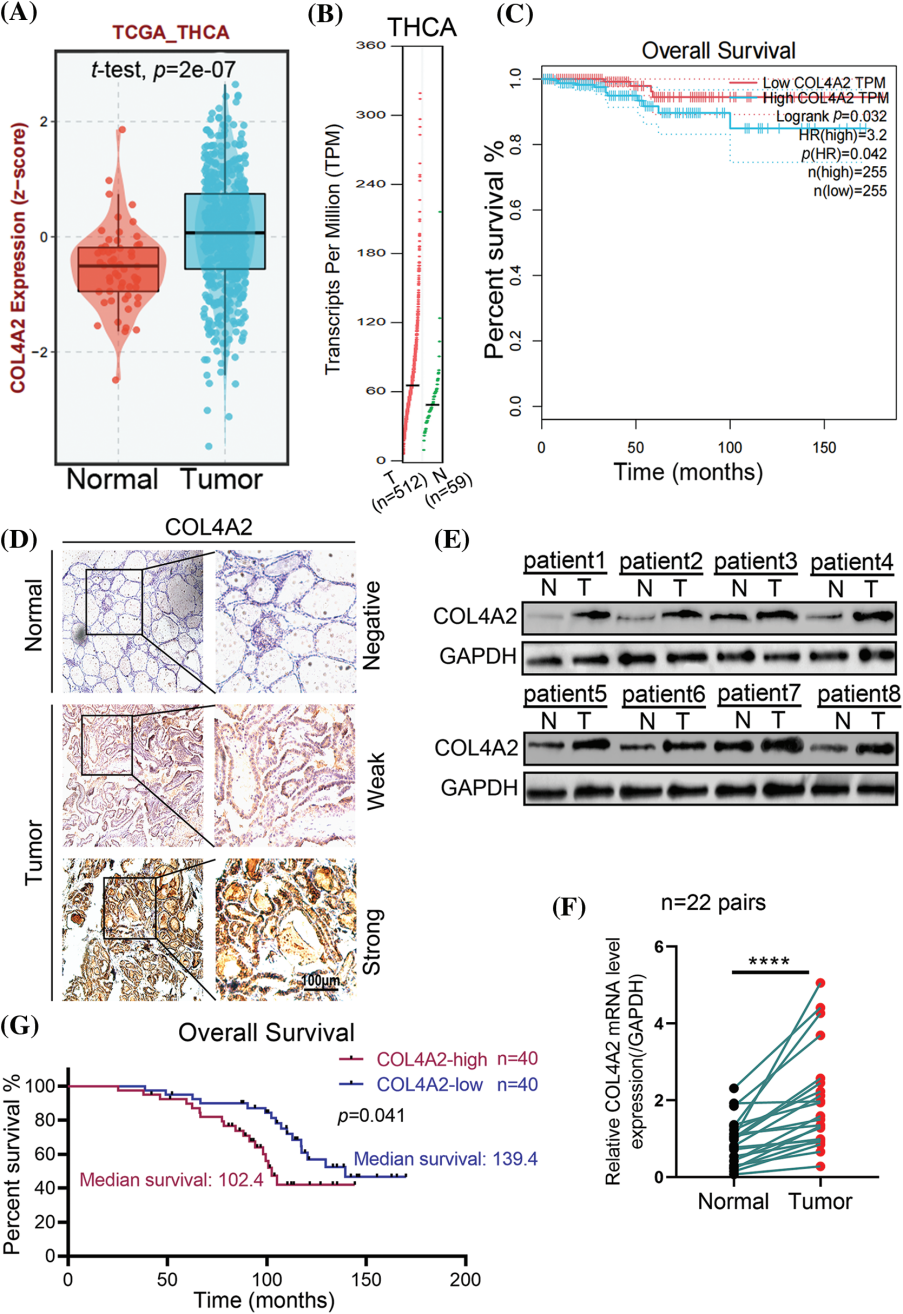
The expression and prognosis of COL4A2 in THCA patients. (A) The expression levels of COL4A2 in THCA tissues are significantly greater than those in adjacent tissues. (B) The transcription level of COL4A2 in THCA tissues was significantly greater than that in adjacent tissues. (C) Kaplan‒Meier curves showing that patients with higher transcriptional levels of COL4A2 in THCA tissue have lower overall survival rates. (D) Immunohistochemistry exhibited COL4A2 expression in tumor tissue was significantly greater than normal, and COL4A2 expression was divided into strong, weak and negative based on differences in COL4A2 expression. Scale bar = 100 μm. (E) Immunoblotting showing COL4A2 expression in 8 pairs of THCA tissues and adjacent normal tissue. (F) Statistical data showing the relative mRNA expression levels in 22 pairs of THCA tissues and adult normal tissues are shown in the line chart. (G) Kaplan–Meier analysis revealed that patients with higher COL4A2 expression in THCA tissue had significantly lower OS than patients with lower COL4A2 expression. The data are expressed as the mean ± SD from 3 repeated experiments. ****p < 0.0001.

After retrieving the expression of COL4A2 in tumor tissues and paracancerous tissues of THCA patients from our TCGA (THCA) dataset, the transcript level and expression level of COL4A2 in THCA tissues were found to be significantly greater than those in paracancerous tissues (Figs. 2A and 2B), and patients with a higher transcript level of COL4A2 in THCA tissues had a lower overall survival rate (Fig. 2C). Subsequently, we performed a protein blot analysis to evaluate the differences in COL4A2 expression between THCA tumor tissues and paired adjacent normal tissues and found that COL4A2 expression was greater in both THCA tumor tissues (Fig. 2D). Despite individual differences in expression levels, COL4A2 expression was significantly greater in THCA tumor tissues than in paired adjacent normal tissues (Fig. 2E), and patients with higher COL4A2 expression in THCA tissues had lower overall survival than patients with lower transcript levels (median survival time 102.4 *vs*. 139.4, *p* = 0.041; Fig. 2F). Immunohistochemistry (IHC) revealed that the expression of COL4A2 in tumor tissue was significantly greater than normal (Fig. 2G), and COL4A2 was divided into high and low-expression (Fig. 2G). Therefore, COL4A2 expression was correlated with survival prognosis in THCA patients.

### The COL4A2 expression level affects the proliferation and activity of THCA cells

**Figure 3:**
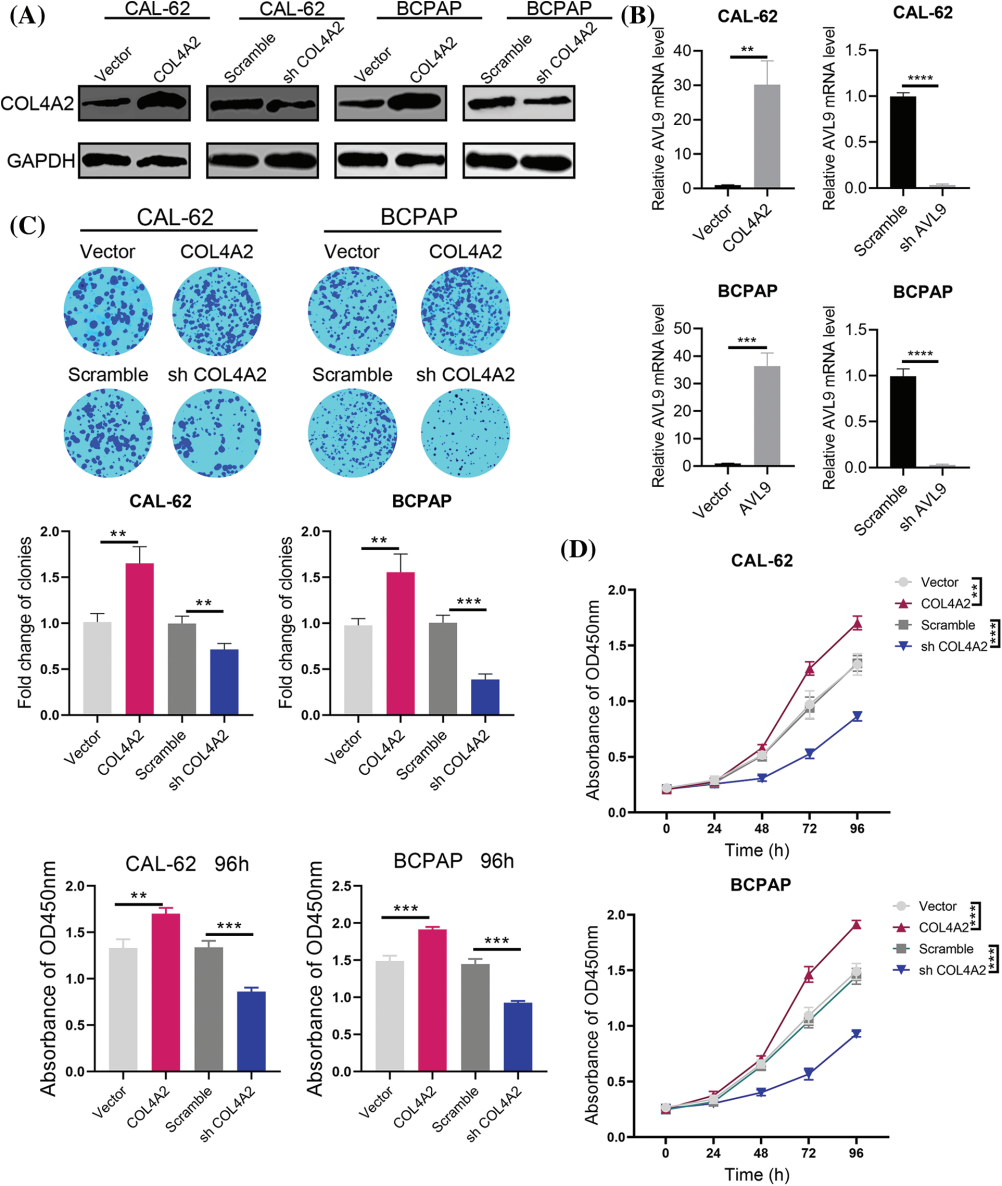
Effect of COL4A2 on THCA cells. (A) Immunoblotting showing the expression of COL4A2 in CAL-62 and BCPAP cells upon the indicated transfection. (B) qPCR analysis of COL4A2 mRNA expression in stable THCA cell lines (CAL-62 and BCPAP cells). (C) Representative images of colony formation assays for the CAL-62 and BCPAP cell lines and quantification of the results. (D) CCK-8 assay of COL4A2-overexpressing cells and COL4A2-silenced cells. The corresponding 96-h-cell index is shown as a histogram. The data are expressed as the mean ± SD from 3 repeated experiments. **p < 0.01; ***p < 0.001; ****p < 0.0001.

We transfected the pLV-COL4A2 lentivirus into CAL-62 and BCPAP cells to increase COL4A2 expression and constructed a shRNA targeting COL4A2 to silence COL4A2 expression in these cells after transfection. The expression of the COL4A2 protein in the COL4A2 group was high, and the expression level of the COL4A2 protein in the shCOL4A2 group was significantly lower than that in the scramble group (Fig. 3A). Similarly, for the relative transcript levels of AVL9 mRNA, the COL4A2 group in the CAL-62 cell line had higher transcript levels than did the vector group, the shCOL4A2 group had lower transcript levels than did the scramble group, the AVL9 group in the BCPAP cell line had higher transcript levels than did the vector group, and the shAVL9 group had lower transcript levels than did the scramble group (Fig. 3B, *p* < 0.05). Colony formation experiments in both cell lines showed that COL4A2-overexpressing cells were able to form more colonies than were COL4A2-silenced cells (fold change in clonies: 1.013 ± 0.093 *vs*. 1.653 ± 0.18, *p* < 0.05; 0.997 ± 0.0815 *vs*. 0.713 ± 0.065, *p* < 0.05; 0.977 ± 0.074 *vs*. 1.557 ± 0.198, *p* < 0.05; and 1.007 ± 0.081 *vs*. 0.387 ± 0.060, *p* < 0.05; Fig. 3C). Similarly, CCK-8 experiments showed that COL4A2-overexpressing cells were more active than COL4A2-silenced cells were (absorbent at OD450 at 96 h: 1.330 ± 0.095 *vs*. 1.703 ± 0.061, *p* < 0.05; 1.340 ± 0.070 *vs*. 0.863 ± 0.040, *p* < 0.05; 1.490 ± 0.072 *vs*. 1.913 ± 0.035, *p* < 0.05; 1.447 ± 0.070 *vs*. 0.927 ± 0.025, *p* < 0.05; Fig. 3D). Therefore, high expression of COL4A2 would promote the growth as well as activity of THCA cells.

### COL4A2 affects THCA cells through the AKT pathway

**Figure 4:**
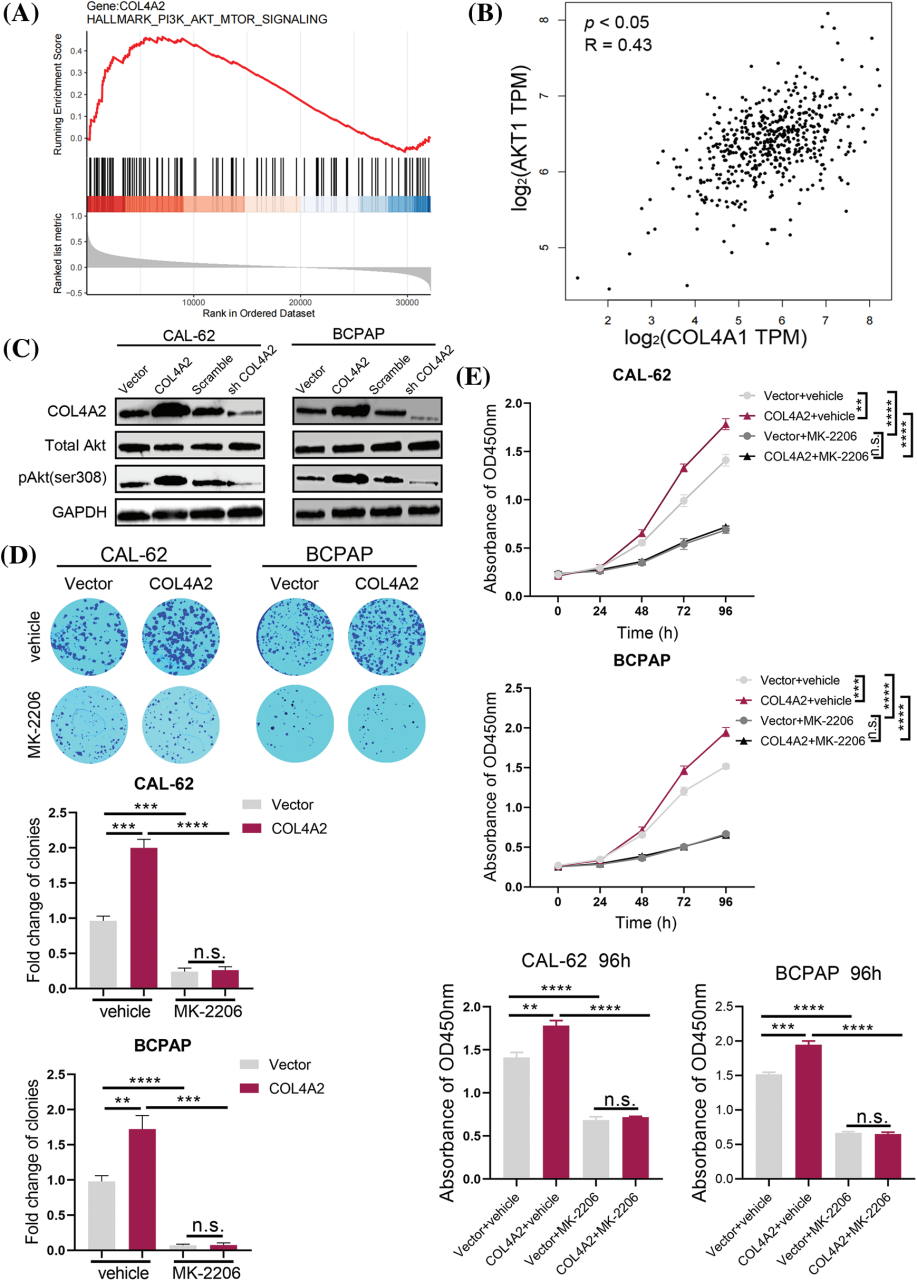
COL4A2 affects THCA cells through the AKT pathway. (A) The TCGA dataset was subjected to GSEA to evaluate COL4A2 expression. We found that the AKT pathway was positively correlated with COL4A2 expression. (B) There was a significant positive correlation between COL4A2 and AKT expression in the TCGA (THCA) cohort. (C) Immunoblotting of fractionated CAL-62 and BCPAP cells after the indicated transfection. (D) Cell cloning ability was analyzed. (E) A CCK-8 assay showed growth of THCA cells treated with MK-2206 and physiological saline. The corresponding 96-h-cell index is shown. The data are expressed as the mean ± SD from 3 repeated experiments. **p < 0.01; ***p < 0.001; ****p < 0.0001. n.s., not significant.

In the course of the above studies, we verified the effect of COL4A2 expression on THCA cells. Next, we investigated the specific downstream mechanisms by which COL4A2 exerts its effects. GSEA was performed, and the AKT transcript level was correlated with the COL4A2 transcript level (*p* < 0.05, R = 0.43; Figs. 4A and 4B). To determine the role of the AKT pathway in the influence of COL4A2 on the growth as well as activity of THCA cells, we performed protein blotting experiments, which showed that an increase in COL4A2 led to an increase in pAKT (ser308), while the total AKT level remained unchanged, indicating activation of the AKT pathway (Fig. 4C). Therefore, COL4A2 mediated the AKT pathway.

Next, we added the AKT pathway blocker MK-2206 and performed the colony formation and CCK-8 assays again. THCA cells had more difficulty forming colonies after the addition of MK-2206, independent of the presence of COL4A2 overexpression (fold change in clonies: 0.963 ± 0.067 *vs*. 2.000 ± 0.121, *p* < 0.05; 0.243 ± 0.050 *vs*. 0.263 ± 0.047, *p* > 0.05; 0.980 ± 0.082 *vs*. 1.723 ± 0.192, *p* < 0.05; 0.073 ± 0.015 *vs*. 0.077 ± 0.031, *p* > 0.05; Fig. 4D). Similarly, CCK-8 assays showed that THCA cells were less active after the addition of MK-2206 (absorbance at OD450 nm at 96 h: 1.410 ± 0.060 *vs*. 1.783 ± 0.057, *p* < 0.05; 0.687 ± 0.035 *vs*. 0.717 ± 0.012, *p* > 0.05; 1.517 ± 0.032 *vs*. 1.947 ± 0.050, *p* < 0.05; 0.667 ± 0.021 *vs*. 0.653 ± 0.029, *p* > 0.05; Fig. 4E). These experimental results suggest that COL4A2 can affect the growth and activity of THCA cells via the AKT pathway and that the AKT pathway deserves further investigation as a therapeutic target for THCA.

### Blocking the AKT pathway of COL4A2 inhibits THCA tumor growth in mice

**Figure 5:**
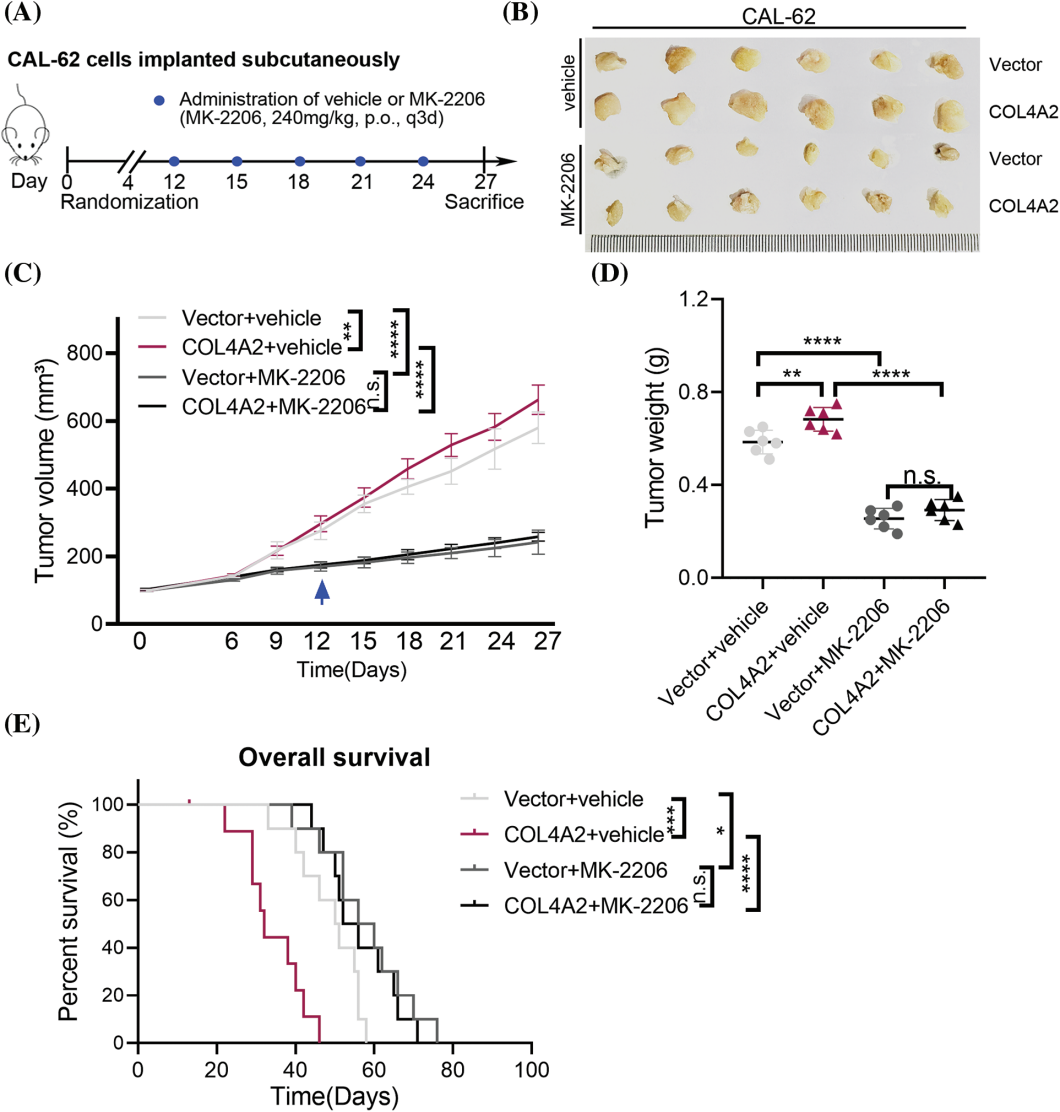
An AKT pathway blocker inhibited THCA tumor growth in mice. (A) Schematic diagram of the mouse experiment. (B) The indicated cell lines were subcutaneously transplanted into nude mice to establish tumors (n = 6 per group). Representative images of the tumors. (C) Repeated measures two-way ANOVA (time × tumor volume) was performed to compare the tumor growth curves among the 4 groups. (D) Tumor weights at the end of the experiment. (E) Kaplan–Meier curves of 4 groups of mice (n = 8 per group). The data are expressed as the mean ± SD from three independent experiments. *p < 0.05; **p < 0.01; ***p < 0.001; ****p < 0.0001 (one-way ANOVA). n.s., not significant.

**FIGURE A2:**
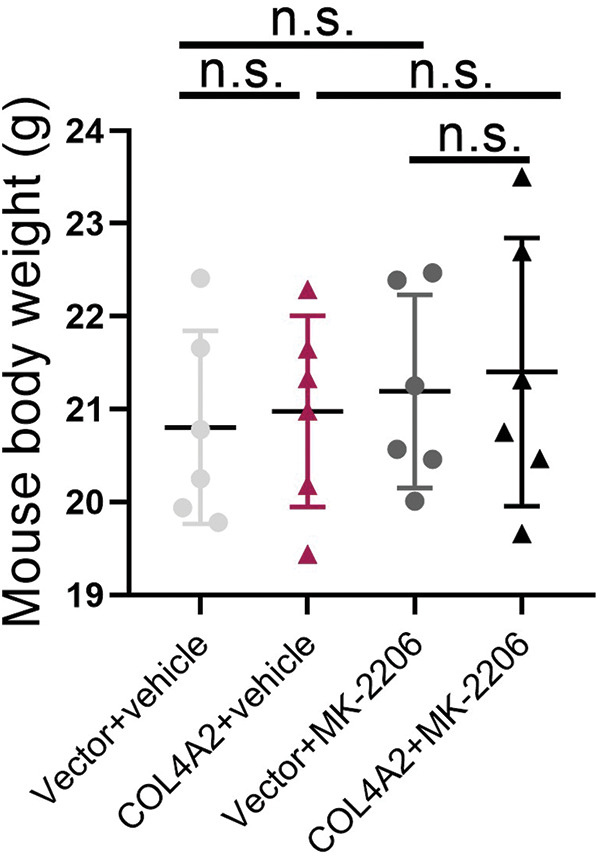
The weight of the animals between the different groups after the *in vivo* experiment. n.s.: not significant.

The above experiments showed that COL4A2 can act on THCA through the AKT pathway. Therefore, a subcutaneous mouse xenograft model was used to test whether MK-2206 could inhibit THCA tumor growth *in vivo*. The tumor volume was measured every 3 days for 6 days after the tumor cells were inoculated; MK-2206 was used to block the AKT pathway, and a saline control group was used (Fig. 5A). The results showed that MK-2206 treatment reduced the tumor volume (Fig. 5B). The tumor volume was measured every 3 days for the duration of the experiment, and it was evident that MK-2206 treatment significantly slowed tumor growth. The tumor volume increased faster in mice not treated with MK-2206 than in mice treated with MK-2206 (Fig. 5C, *p* < 0.05). Moreover, MK-2206 treatment also reduced tumor weight (Fig. 5D, *p* < 0.05). Moreover, in a parallel survival trial, MK-2206 treatment significantly extended the survival of mice (Fig. 5E). Of course, we could also see that there is no statistical difference in the weight of the animals between the different groups (Fig. A2, *p* > 0.05, separately). Our findings suggest that the AKT pathway may be a target for inhibiting the promotion of THCA by COL4A2, which may provide an alternative option for treating THCA with high COL4A2 expression levels.

## Discussion

Thyroid cancer is diagnosed in 3.4% of all cancer patients each year. Over the last 40 years, worldwide, the incidence of this disease has been on the rise due to increased screening and environmental and lifestyle changes, which have caused the incidence of THCA to triple in the last 30 years [11]. Currently, treatment options for most thyroid cancer patients include total thyroidectomy, removal of affected cervical lymph nodes, radioactive iodine (RAI) therapy to ablate residual or metastatic foci of the thyroid, and TSH suppression with l-thyroxine [12]. In this study, we discussed the importance of developing new therapeutic targets for thyroid cancer.

With these treatments, the majority of thyroid cancer patients have a good prognosis, with a survival rate of 98% [13]. However, some tumors become more aggressive, and some undergo a progressive process of dedifferentiation, decreasing the production of thyroglobulin and concentrated iodine and decreasing the response to RAI [14]. To identify patients with a progressive disease course, scholars have proposed various prognostic factors and clinical scores, mainly age, histological variant, initial extent of disease and size of the primary tumor [15]. However, these prognostic factors have several limitations, making it difficult to identify patients with a poor prognosis [16]. Therefore, in the case of thyroid cancer, a better understanding of the gene expression products that promote the progression of this disease may be key to adapting its treatment and management.

To improve the cure rate of THCA, more effective targets need to be identified for cancer therapy [17]. ECM is a key component of the tumor microenvironment [18]. As a member of the ECM, COL4A2 may influence tumor progression through ECM [19]. On the other hand, collagen itself, as a secreted protein, has great potential as a tumor biomarker and has been demonstrated to be highly sensitive and specific for a variety of tumors [8]. For cancer, a highly malignant and metastatic tumor, finding early tumor evidence from collagen can aid early diagnosis [20]. Collagen is a promising biomarker for early cancer detection.

After a series of bioinformatics studies, COL4A2 was found to be highly expressed in human cancer tissues, and we also found a correlation between prognosis, clinical features and COL4A2 expression. The collagen family can modulate extracellular matrix (ECM) receptor interactions in tumor and local adhesion pathways and therefore affect cancer progression and metastasis [21], and the prognostic impact of collagen on cancer has been widely revealed [22]. A previous study showed that seven collagen genes (COL1A2, COL4A1, COL6A2, COC6A1, COL4A2, and COL11A1) were highly expressed in gastric cancer tissues, consistent with our findings [23].

In subsequent experiments, we found a correlation between COL4A2 expression and the growth and activity of THCA cells. Our findings suggest that COL4A2 is vital in THCA progression. Next, on the basis of our raw letter analysis, we investigated the role of the AKT pathway in COL4A2-related thyroid cancer development. The AKT pathway is a well-established oncogenic pathway. The results indicated that the AKT pathway of COL4A2 contributed to the growth of THCA cells, suggesting that this pathway could be used as an alternative for the treatment of THCA patients with high COL4A2 expression. High expression of COL4A2 promotes abnormal activation of the AKT axis, which leads to tumor proliferation. As a classical inhibitor of the AKT pathway, MK-2206 significantly inhibited the proliferation of COL4A2-overexpressing cell lines.

## Conclusions

The conclusions of this study need to be widely verified by multi-center and large sample clinical data, and the related mechanisms also need to be further explored in the signaling pathway.

Here, we demonstrated that COL4A2 are high expression in THCA cells, that COL4A2 expression affects overall survival, and that blocking the AKT pathway of COL4A2 inhibits the proliferation of THCA cells. Our findings also suggested that COL4A2 inhibitors may be used to develop new therapies for thyroid cancer. In conclusion, we innovatively revealed a new regulatory mechanism of AKT pathway activation and suggested a more specific therapeutic approach for THCA. For instance, we can evaluate the therapeutic effect of MK-2206 by detecting the expression level of COL4A2 and try to use it to boost the therapeutic efficacy in patients with high COL4A2 expression. Therefore, we believe that COL4A2 can be used as a new research target.

## Data Availability

The dataset supporting the conclusions of this article is included within the article.
